# Cardiac expression of the CREM repressor isoform CREM-IbΔC-X in mice leads to arrhythmogenic alterations in ventricular cardiomyocytes

**DOI:** 10.1007/s00395-016-0532-y

**Published:** 2016-01-27

**Authors:** J. S. Schulte, E. Fehrmann, M. A. Tekook, D. Kranick, B. Fels, N. Li, X. H. T. Wehrens, A. Heinick, M. D. Seidl, W. Schmitz, F. U. Müller

**Affiliations:** Institute of Pharmacology and Toxicology, University of Münster, Domagkstr. 12, 48149 Münster, Germany; Department of Molecular Physiology and Biophysics, Medicine (Cardiology), and Pediatrics, Cardiovascular Research Institute, Baylor College of Medicine, Houston, TX USA

**Keywords:** Transcription factor CREM, Arrhythmia, Remodeling, NCX

## Abstract

Chronic β-adrenergic stimulation is regarded as a pivotal step in the progression of heart failure which is associated with a high risk for arrhythmia. The cAMP-dependent transcription factors cAMP-responsive element binding protein (CREB) and cAMP-responsive element modulator (CREM) mediate transcriptional regulation in response to β-adrenergic stimulation and CREM repressor isoforms are induced after stimulation of the β-adrenoceptor. Here, we investigate whether CREM repressors contribute to the arrhythmogenic remodeling in the heart by analyzing arrhythmogenic alterations in ventricular cardiomyocytes (VCMs) from mice with transgenic expression of the CREM repressor isoform CREM-IbΔC-X (TG). Patch clamp analyses, calcium imaging, immunoblotting and real-time quantitative RT-PCR were conducted to study proarrhythmic alterations in TG VCMs vs. wild-type controls. The percentage of VCMs displaying spontaneous supra-threshold transient-like Ca^2+^ releases was increased in TG accompanied by an enhanced transduction rate of sub-threshold Ca^2+^ waves into these supra-threshold events. As a likely cause we discovered enhanced NCX-mediated Ca^2+^ transport and NCX1 protein level in TG. An increase in *I*_NCX_ and decrease in *I*_to_ and its accessory channel subunit KChIP2 was associated with action potential prolongation and an increased proportion of TG VCMs showing early afterdepolarizations. Finally, ventricular extrasystoles were augmented in TG mice underlining the in vivo relevance of our findings. Transgenic expression of CREM-IbΔC-X in mouse VCMs leads to distinct arrhythmogenic alterations. Since CREM repressors are inducible by chronic β-adrenergic stimulation our results suggest that the inhibition of CRE-dependent transcription contributes to the formation of an arrhythmogenic substrate in chronic heart disease.

**Electronic supplementary material:**

The online version of this article (doi:10.1007/s00395-016-0532-y) contains supplementary material, which is available to authorized users.

## Introduction

Heart failure (HF) is characterized by cardiac dysfunction but likewise associated with a high risk for life threatening arrhythmias especially in mild to moderate disease stages. Up to 50 % of HF associated deaths are attributed to the “sudden cardiac death” caused by arrhythmia [1, 15]. During the development of HF the heart undergoes an extensive remodeling process which not only results in impairment of cardiac function but also in the formation of an arrhythmogenic substrate. In cardiomyocytes these alterations include action potential (AP) prolongation along with the reduction of potassium currents (*I*_to_, *I*_Ks_, *I*_Kr_, *I*_K1_) facilitating the occurrence of early afterdepolarizations (EADs) during AP repolarization. Furthermore, alterations of the intracellular Ca^2+^ homeostasis lead to an enhanced occurrence of spontaneous Ca^2+^ releases which could be translated into delayed afterdepolarizations (DADs) by the electrogenic NCX current (*I*_NCX_) as a trigger for arrhythmia [30, 31, 39]. However, the molecular mechanisms leading to this remodeling are poorly understood [35].

The transcription factors cAMP-responsive element binding protein (CREB) and cAMP-responsive element modulator (CREM) bind to cAMP response elements (CREs) in the promoter regions of target genes and mediate transcriptional regulation, inter alia, in response to stimulation of cAMP-dependent signaling pathways [2]. Catecholamines acting as agonists of such pathways are chronically elevated in HF patients and are regarded as a pivotal step in the progression of the disease. There is evidence that major ventricular arrhythmias are associated with sustained cardiac sympathetic activation in HF [16, 23], and β-blockers have been proven to reduce the risk for sudden cardiac death [11, 13, 19]. The CREM gene encodes several structurally related isoforms, which function as activators or repressors of transcription. The small CREM repressor isoform ICER (inducible cAMP early repressor) is up-regulated in human HF [10]. ICER proteins are rapidly induced by cAMP elevation and are strong repressors of CRE-mediated transcription [24]. We recently reported that β-adrenergic stimulation by isoproterenol leads to the upregulation of a novel CREM repressor isoform, small ICER (smICER), beside ICER in the mouse heart [36]. The CREM repressor isoform CREM-IbΔC-X was first identified in failing human heart tissue [25]. Transgenic mice with heart-directed expression of CREM-IbΔC-X spontaneously develop atrial fibrillation (AF) [7, 17, 18, 27] and AF susceptibility in human is inter alia associated with decreased expression of CREB target genes [9]. Furthermore, inactivation of the transcriptional activator CREB leads to AP prolongation with downregulation of the transient outward current (*I*_to_) and its underlying channel subunit Kv4.2 in ventricular cardiomyocytes (VCMs) [34]. These results led to the hypothesis that the inhibition of cAMP-dependent transcription by the induction of small CREM repressor isoforms participates in an arrhythmogenic remodeling in cardiomyocytes. Here, we tested this hypothesis by investigating arrhythmogenic alterations in VCMs, their molecular causes, and in vivo consequences in CREM-IbΔC-X transgenic mice.

## Methods

Detailed methods are available in the data supplement.

### Experimental animals

Mice with heart-directed expression of CREM-IbΔC-X (TG) [7, 17, 18, 27] and wild-type littermates (CTL) were studied at 16–21 weeks of age if not indicated otherwise. All experiments were in accordance with the local animal welfare authorities, conform to the Directive 2010/63/EU of the European Parliament and were approved by regional authorities (LANUV; North Rhine-Westphalia, Germany; permit 84-02.04.2011.A179).

### Cell isolation

Primary adult VCMs were isolated as described previously by collagenase/protease digestion in a Langendorff apparatus [34].

### Patch clamp

Action potentials and membrane currents were recorded using the perforated patch technique with amphotericin B. APs and L-type Ca^2+^ currents (*I*_Ca,L_) were evoked as described [34]. Potassium currents (*I*_K,total_) were evoked by a single step protocol from −40 to +60 mV from a holding potential (HP) of −80 mV for 25 s to achieve complete inactivation of transient components of *I*_K,total_. *I*_NCX_ was elicited by caffeine application (10 mM) as described [32].

### Calcium imaging

Intracellular Ca^2+^ (Ca^2+^_i_) transients and sarcomere shortening were recorded simultaneously after loading VCMs with Indo-1/AM (0.5 Hz stimulation, 22–24 °C) under basal conditions and during superfusion with 1 µM isoproterenol. For the detection of spontaneous Ca^2+^ releases (sCaRs) cardiomyocytes were prestimulated at 1 and 2 Hz for 30 s each followed by a 90 s stimulation pause in which sCaRs were counted. Separation of SERCA2a, NCX and the sarcolemmal Ca^2+^-ATPase (PMCA) was performed in VCMs loaded with Fluo-4/AM as described [20].

### Immunoblotting and quantitative real-time RT-PCR

Immunoblotting and quantitative real-time RT-PCR were performed on ventricular homogenates as described previously [34, 36].

### ECG recordings

ECGs were recorded from anesthetized old (19–21 weeks of age) and young mice (5–7 weeks of age, before onset of AF). Baseline ECGs were recorded for 10 min followed by an additional 10 min (old mice) or 20 min (young mice) recording-phase after intraperitoneal injection of isoproterenol (2 mg/kg body weight).

### Chronic isoproterenol treatment of CTL mice

Osmotic minipumps (model 2001; Alzet, Cupertino, USA) were implanted in CTL mice (anesthesia: isoflurane/N_2_O 1.5–2 %/60 %) for continuous application of isoproterenol (3 mg/kg/day) according to the manufacturer’s instructions.

### Statistical analysis

If not indicated otherwise, data are presented as mean ± SEM or box plots (box 25th–75th percentile, whiskers: 10th–90th percentile, horizontal line: median, square: mean). The number of experiments is reported as *n* = cells/animals or *n* = number of independent samples. Statistical analysis was performed using rank-sum-test, Student’s *t* test or Chi square test where adequate. Relative expression ratios of mRNAs were calculated using the ΔΔ*C*_T_ method with REST software [38].

## Results

### Overexpression of CREM-IbΔC-X leads to an increased proportion of cardiomyocytes with spontaneous transient-like Ca^2+^ releases

**Fig. 1:**
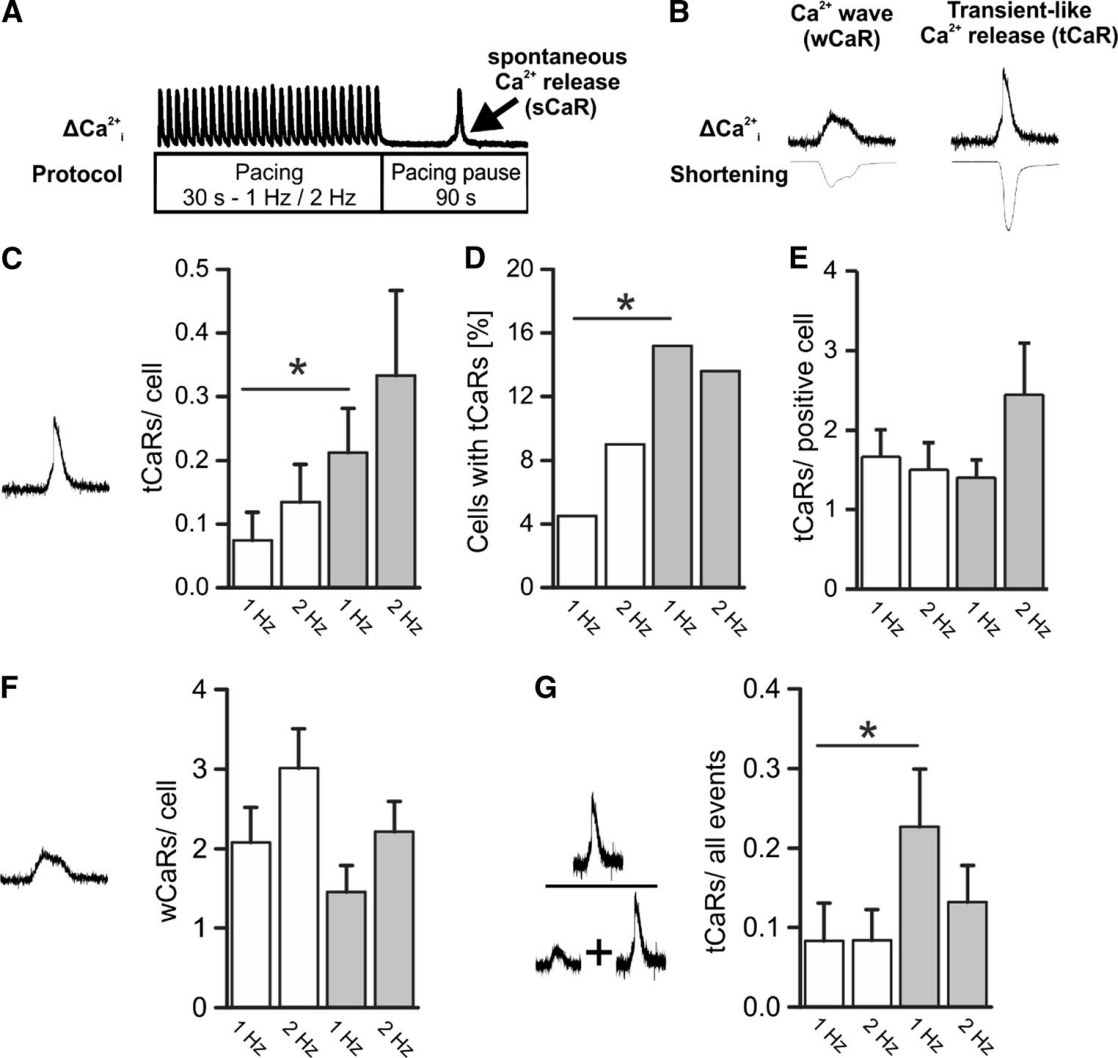
**a** Field stimulation protocol to provoke spontaneous Ca^2+^ events in Indo-1/AM loaded VCMs during a 90 s stimulation pause after a 30 s lasting 1 and 2 Hz stimulation phase. Two event types were detected as displayed in **b**: sub-threshold Ca^2+^ waves (wCaR) and supra-threshold Ca^2+^ releases (transient-like, tCaR). The ratio tCaR/all measured cells was increased in TG VCMs (**c**) due to a higher proportion of VCMs showing this event type (**d**) while the tCaR-frequency in tCaR-positive VCMs was not different between groups (**e**). The rate of Ca^2+^ waves/VCM was unaltered between groups (**f**) while the transduction of wCaR into tCaR expressed by the ratio tCaR/(wCaR + tCaR) was increased in TG (**g**) (*white bars* CTL, *n* = 67/11; *grey bars* TG, *n* = 66/12; *p < 0.05 vs. CTL)

Macroscopic spontaneous Ca^2+^ releases which are regarded as a trigger for arrhythmia were provoked in VCMs of TG and CTL mice by a stimulation-pause-protocol as displayed in Fig. 1a. In the respective pacing pauses we observed sub-threshold Ca^2+^ waves (wave-type, wCaR) and supra-threshold, transient-like Ca^2+^ releases (tCaR) arising from a wCaR (Fig. 1b). Averaged over all observed VCMs tCaRs occurred more than twice as often (Fig. 1c) and in tendency earlier (Suppl. Fig. 1) in TG VCMs while the occurrence of wCaRs was not different between groups (Fig. 1f). The mean increase in tCaR was due to an increased recruitment of tCaR-positive VCMs (Fig. 1d) and not due to an increased tCaR-frequency in these event-positive VCMs (Fig. 1e). Interestingly, the ratio tCaR/all detected events reflecting the transduction rate of wCaRs into supra-threshold tCaRs was increased in TG VCMs (Fig. 1g) pointing to a mechanism which facilitates the triggering of APs by Ca^2+^ releases.

**Fig. 2:**
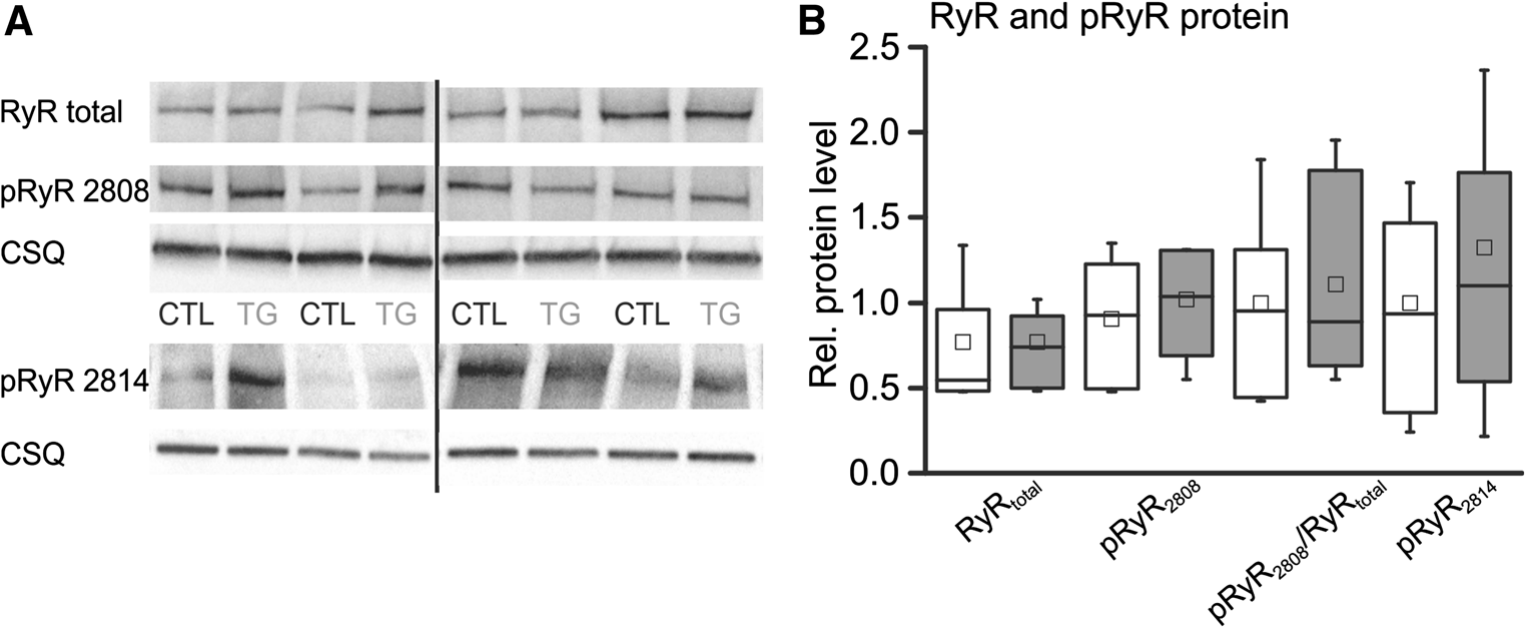
**a** Representative immunoblots and **b** relative protein levels for total RyR2, Ser2808 and Ser2814 phosphoforms normalized to CSQ. Protein levels were not significantly different between groups, albeit along a noticeable sample to sample variation (*white boxes* CTL, *grey boxes* TG, *n* = 12/group)

In line with the view of an unaltered Ca^2+^ release frequency but facilitated transduction of wCaRs into tCaRs in TG the levels of total RyR2 protein and its phosphorylated forms (Ser2808/PKA, Ser2814/CaMKII) in ventricular homogenates from TG and CTL mice (Fig. 2a, b) were not significantly different between groups, though we detected a noticeable sample to sample variation.

### Altered Ca^2+^ homeostasis but no increase in SR Ca^2+^ load in TG VCMs

**Fig. 3:**
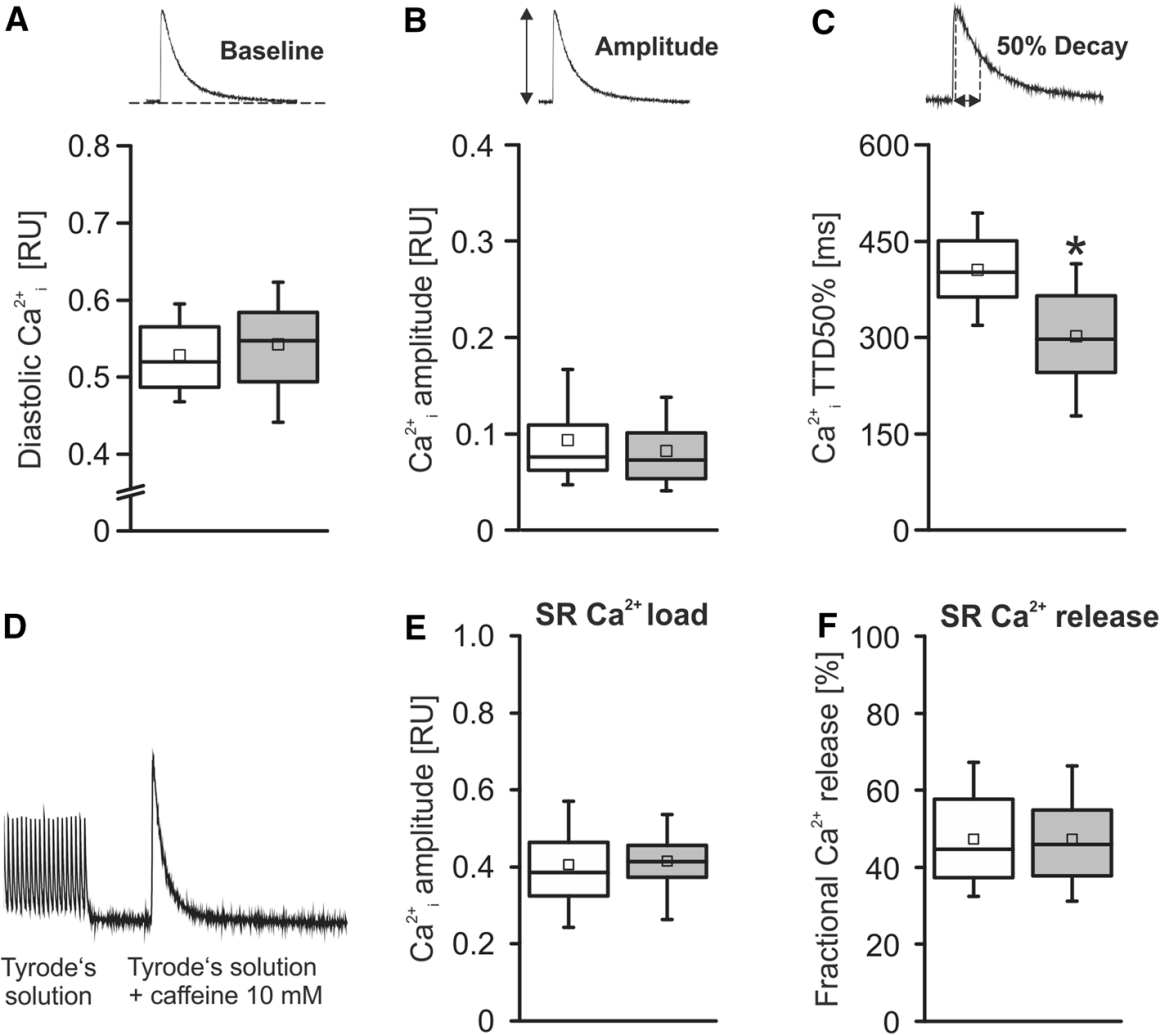
**a** Diastolic Ca^2+^_i_ and **b** the Ca^2+^_i_ transient amplitude were unaltered between groups. **c** The Ca^2+^_i_ transient decay was accelerated in TG myocytes (TTD50 % in ms) pointing to altered Ca^2+^ transport/extrusion mechanisms in TG (*white boxes* CTL basal, *n* = 99/10; *grey boxes* TG basal, *n* = 97/10; *p < 0.05 vs. CTL). Rapid caffeine application to Indo-1/AM loaded VCMs after prestimulation as displayed in **d** was performed to assess **e** SR Ca^2+^ load and **f** fractional SR Ca^2+^ release which were unaltered between groups (*white boxes* CTL, *n* = 39/5; *grey boxes* TG, *n* = 33/4)

Next we examined intracellular Ca^2+^ homeostasis in TG VCMs for arrhythmogenic alterations. Diastolic Ca^2+^_i_ was unaltered between groups (Fig. 3a). The Ca^2+^_i_ transient amplitude was likewise unaltered in TG vs. CTL (Fig. 3b), whereas the decay of the Ca^2+^_i_ transient was accelerated in TG VCMs by 16–22 % as shown by a reduced time to 50 % decay of peak (TTD50 %, Fig. 3c) suggesting an altered NCX- or SERCA2a-mediated Ca^2+^ transport in TG VCMs. The accelerated transient decay in TG was not associated with an altered SR Ca^2+^ load nor altered fractional Ca^2+^ release between groups as determined in Indo-1/AM loaded VCMs (Fig. 3d–f).

### Ca^2+^ transport rates of SERCA2a and NCX are increased in TG

**Fig. 4:**
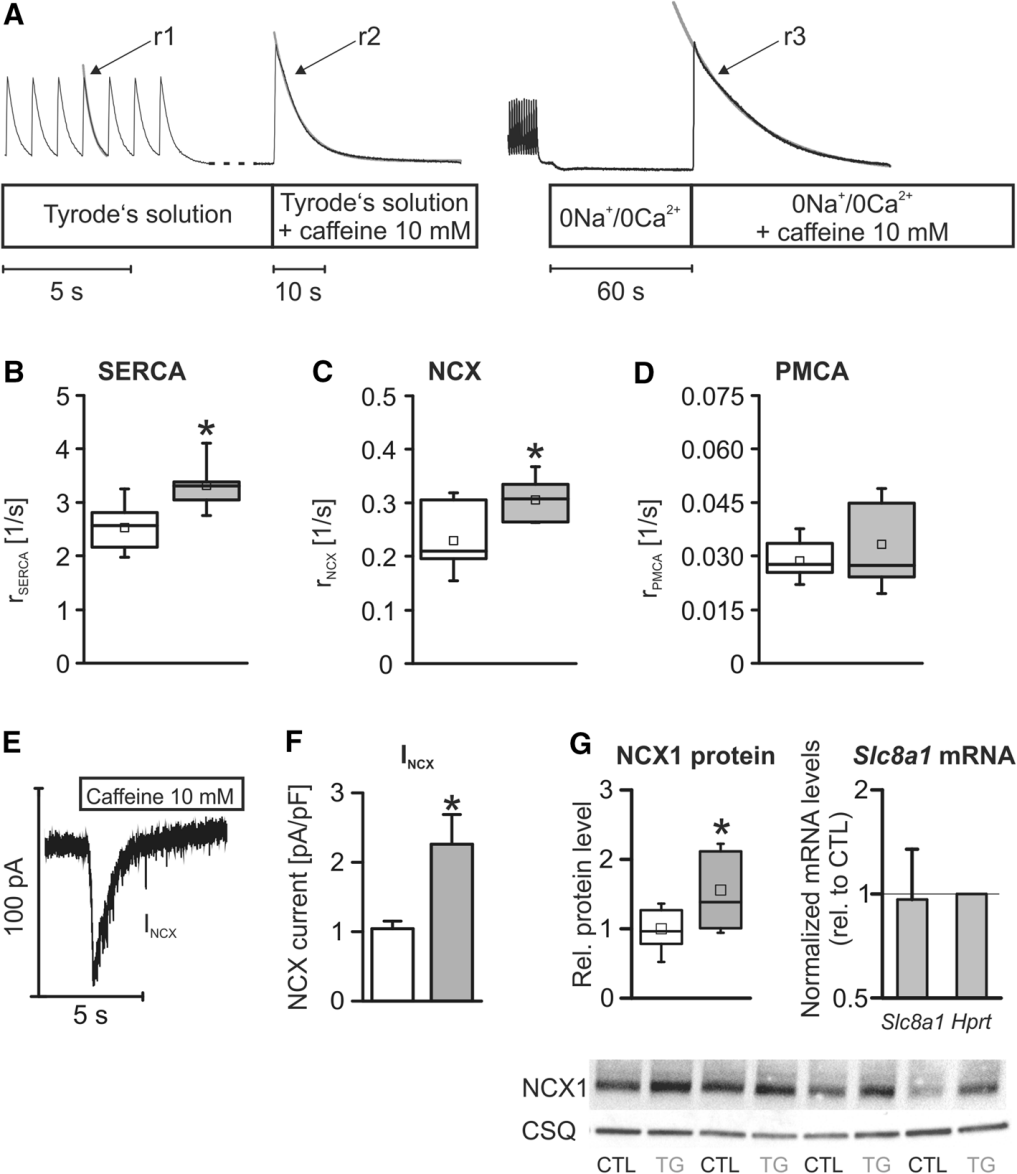
**a** Transport rates for SERCA2a, NCX and the PMCA were determined by exponential fitting to the decay phases of electrically evoked Ca^2+^ transients (*r*_1_ = SERCA + NCX + PMCA), caffeine induced Ca^2+^ transients (*r*_2_ = NCX + PMCA) and caffeine induced Ca^2+^ transients with NCX-block by 0Na^+^/0Ca^2+^-solution (*r*_3_ = PMCA). The SERCA2a transport rate (**b**) (r_SERCA_ = *r*_1_ − *r*_2_) and the NCX transport rate (**c**) (r_NCX_ = *r*_2_ − *r*_3_) were increased in TG VCMs while the PMCA transport rate (**d**) (*r*_PMCA_ = *r*_3_) was unaltered between groups (*white boxes* CTL, *n* = 22–53/6; *grey boxes* TG, *n* = 30–70/6). **e** Original registration of a caffeine induced *I*_NCX_ in a patch clamp experiment. *I*_NCX_ was likewise increased in TG myocytes (**f** peak *I*_NCX_ in pA/pF; *white bar* CTL, *n* = 11/3; *grey bar* TG, *n* = 11/3) along elevated NCX1 protein levels (**g**) (*n* = 12/group) as illustrated by representative immunoblots, whereas *Slc8a1* mRNA levels were not different between groups (*p < 0.05 vs. CTL)

Changes in SERCA2a and NCX both may be arrhythmogenic. Thus, we determined the Ca^2+^ extrusion rate of the Ca^2+^ extrusion proteins SERCA2a, NCX and PMCA in Fluo-4/AM loaded VCMs by the protocol outlined in Fig. 4a to elucidate the cause for the accelerated decay phase of the Ca^2+^_i_ transient in TG VCMs. Calculating the respective Ca^2+^ transport rates r_SERCA_, r_NCX_ and r_PMCA_ revealed an increased r_SERCA_ and r_NCX_ by 39 and 42 %, respectively, and unaltered r_PMCA_ in TG VCMs (Fig. 4b–d), well explaining the observed accelerated Ca^2+^_i_ transient decay in TG myocytes. The increased r_SERCA_ went along with an elevation of SERCA2a protein levels in TG ventricular homogenates as described before [27] and confirmed by immunoblotting (Suppl. Fig. 2). In summary, overexpression of CREM-IbΔC-X led not only to an enhanced SERCA2a- but also NCX-mediated Ca^2+^ transport in VCMs.

### *I*_NCX_ and NCX1 protein levels are increased in TG

We next validated the increased r_NCX_ by directly measuring the NCX current (*I*_NCX_) in patch clamp experiments. As displayed in Fig. 4e, f the caffeine induced *I*_NCX_ was increased in TG VCMs on average by 117 % vs. CTLs. The enhanced r_NCX_ and *I*_NCX_ went along with an increased relative NCX1 protein level to 160 % of CTLs (*p* < 0.05) in TG ventricular homogenates as assessed by immunoblotting (Fig. 4g). However, we were not able to detect any differences in *Slc8a1*/NCX1 mRNA levels between groups by real-time RT-PCR. Thus, the enhanced r_NCX_ and *I*_NCX_ in TG VCMs goes along with an increase in NCX1 protein level without contemporaneous transcriptional alterations.

### Increased NCX in TG is associated with AP prolongation and EADs

**Fig. 5:**
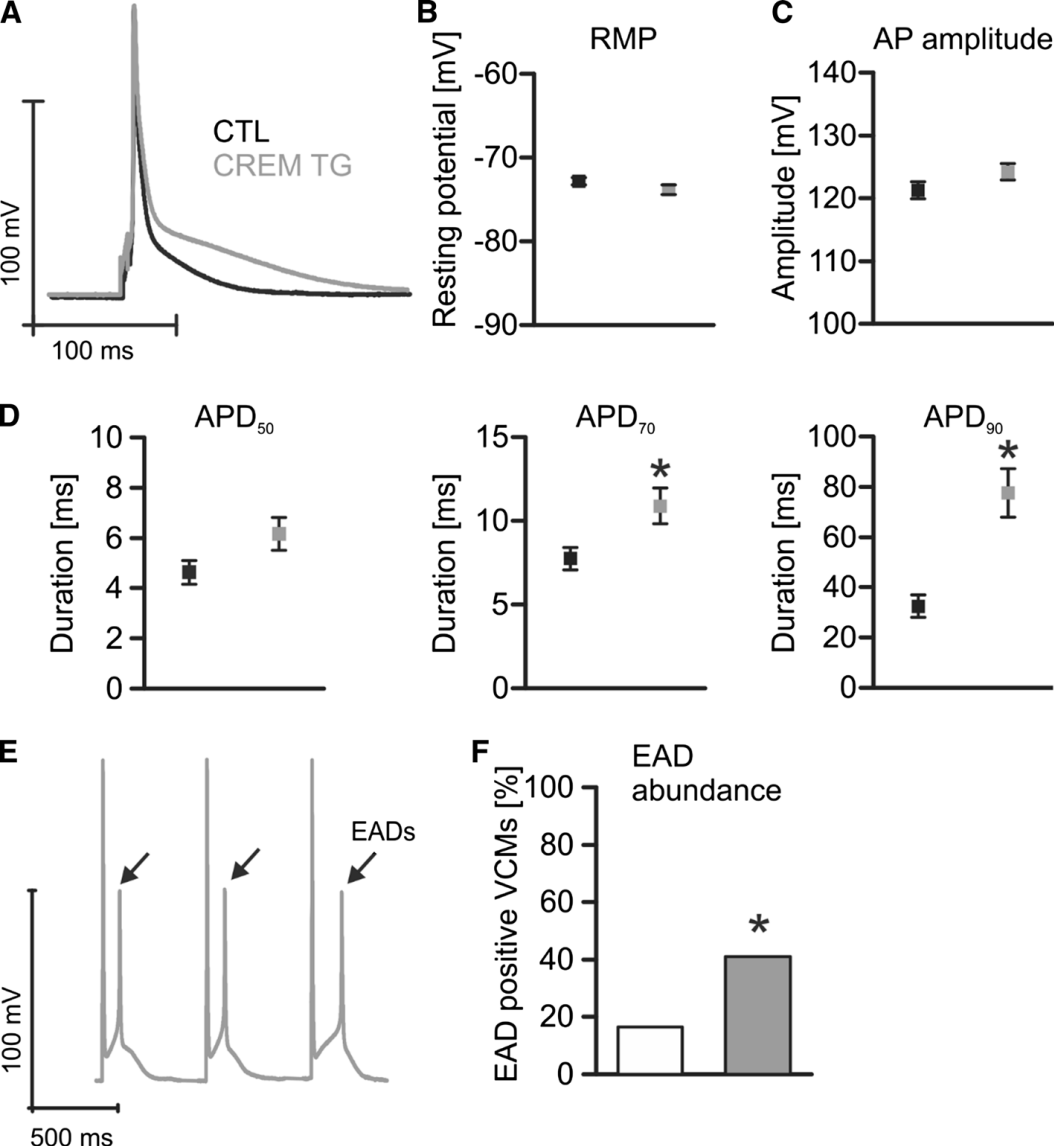
Superimposed representative APs illustrate AP prolongation in TG myocytes (**a**). The resting membrane potential (RMP) (**b**) and AP amplitude (**c**) were unaltered between groups. AP duration until 70 and 90 % repolarization (APD_70,90_) was increased in TG VCMs while APD_50_ was prolonged only in tendency (**d**) (*black* CTL, *n* = 35/13; *grey* TG, *n* = 36/13). **e** Original registration of TG APs with EADs. AP prolongation in TG went along with an increased proportion of VCMs with EADs (**f**) (*p < 0.05 vs. CTL)

Since an increased *I*_NCX_ might prolong the AP we recorded APs from TG and CTL VCMs. APs from TG VCMs were actually prolonged vs. CTLs. As displayed by the representative recordings in Fig. 5a, AP duration at 70 and 90 % repolarization was increased in TG VCMs (APD_70_: 141 % and APD_90_: 239 % of CTLs) while APD_50_ (Fig. 5d) was only increased by tendency. At the same time neither the resting membrane potential (RMP, Fig. 5b) nor the AP amplitude (Fig. 5c) were different between groups. The observed AP prolongation in TG VCMs went along with a doubled proportion of VCMs showing EADs (Fig. 5e, f).

### Overexpression of CREM-IbΔC-X leads to potassium current remodeling

**Fig. 6:**
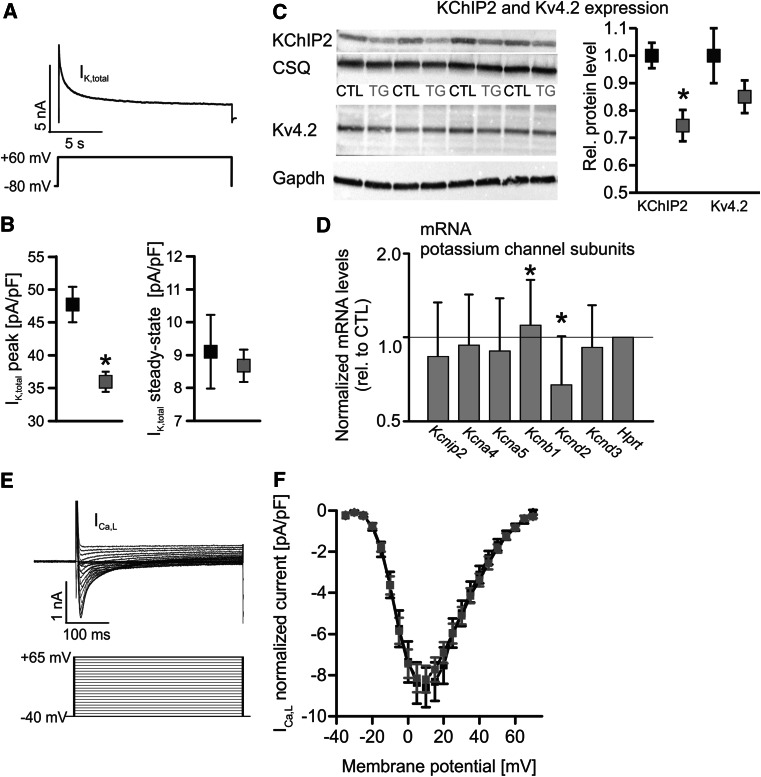
**a** *I*_K,total_ peak and steady-state amplitudes were determined by a single 25 s voltage pulse to +60 mV (HP −80 mV) for complete transient current inactivation. **b** *I*_K,total_ peak amplitude was reduced by 25 % in TG VCMs whereas the steady-state amplitude was unaltered between groups (*black* CTL, *n* = 14/7; *grey* TG, *n* = 23/9). **c** As illustrated by representative immunoblots KChIP2 protein was down-regulated in TG ventricular homogenates (relative protein level, KChIP2 normalized to CSQ, Kv4.2 normalized to Gapdh, *n* = 5–6). **d** Relative expression levels of mRNAs encoding major potassium channel subunits in ventricular homogenates from TG and CTL mice. The relative expression level of *Kcnd2*/Kv4.2 (*I*_to_) was decreased whereas *Kcnb1*/Kv2.1 (*I*_Kslow2_) was increased in TG [relative mRNA expression level normalized to *Hprt* (hypoxanthine-guanine phosphoribosyltransferase)]. mRNA levels encoding *Kcnip2*/KChIP2 (*I*_to_, accessory subunit), *Kcna4*/Kv1.4 (*I*_to_), *Kcna5*/Kv1.5 (*I*_Kslow1_), and *Kcnd3*/Kv4.3 (*I*_to_) were unaltered between groups. **e** A classic step protocol was used to evoke *I*_Ca,L_ (−40 to +65 mV, Δ5 mV, 400 ms duration, HP −80 mV, 1 Hz stimulation frequency). Sodium currents were inactivated by a 200 ms prepulse before each step (truncated in the figure). The calculated *I*–*V*-relationship was not different between groups (**f**) (*p < 0.05 vs. CTL)

Next we examined whether alterations in other major currents (*I*_K_, *I*_Ca,L_) contribute in addition to the increased *I*_NCX_ to the AP prolongation observed in CREM-IbΔC-X TG mice. As displayed in Fig. 6a, b the *I*_K,total_ peak amplitude was reduced by 25 % in TG VCMs whereas the steady-state amplitude was unaltered between groups pointing to a reduction of *I*_to_ in TG VCMs. The *I*_K,total_ peak amplitude reduction was accompanied by a 26 % reduction in KChIP2 protein levels, an important accessory *I*_to_ underlying subunit [12], while the main pore forming subunit in mice Kv4.2 was only reduced by tendency in TG ventricular homogenates (Fig. 6c).

However, the mRNA level of *Kcnd2*/Kv4.2 was reduced in TG ventricular homogenates while the *Kcnip2*/KChIP2 mRNA level was unaltered between groups (Fig. 6d). mRNA levels of the potassium channel subunits *Kcnd3*/Kv4.3, *Kcna5*/Kv1.5 and *Kcna4*/Kv1.4 were not altered between groups, except an increase of *Kcnb1*/Kv2.1 mRNA in TG ventricular homogenates.

The *I*–*V*-relationship of *I*_Ca,L_ (Fig. 6f) was not different between groups excluding this current as an explanation for the prolonged APs in TG VCMs. Hence, the reduction of *I*_K,total_ in TG VCMs is most likely due to a reduction of KChIP2 leading to a decrease of *I*_to_ which will contribute to the AP prolongation in TG VCMs in addition to the increase in *I*_NCX_.

### No hypertrophy of VCMs in CREM-IbΔC-X mice

The measured size of VCMs from the same preparations used for the single cell studies was not different between groups as determined by wide-field microscopy. VCM capacities derived from the patch clamp experiments were likewise unaltered between groups excluding cellular hypertrophy in CREM-IbΔC-X VCMs (Suppl. Fig. 3).

### TG mice display an increase in ventricular extrasystoles

**Fig. 7:**
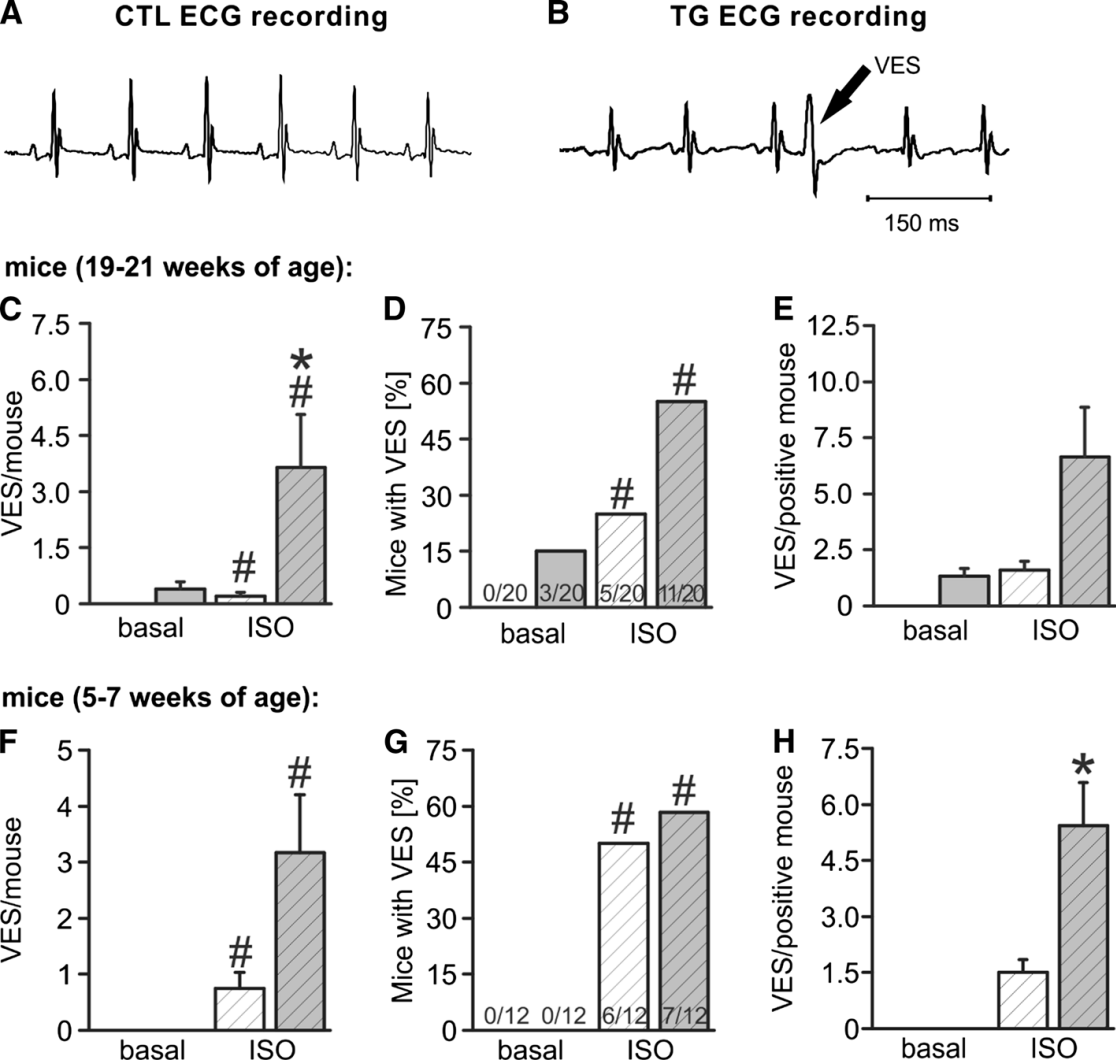
Representative ECG recording of **a** a WT mice and **b** a TG mice displaying a VES. **c** Mean VES/all measured mice, **d** proportion of mice with VES and **e** mean VES/positive mouse in older mice (19–21 weeks, *n* = 20), **f**–**h** in young mice (5–7 weeks, *n* = 12), respectively. Older TG mice had on average more VES/mouse after application of isoproterenol (ISO) because both proportion of mice showing VES and VES frequency in positive mice were tendentially increased. In younger TG mice before the onset of AF the VES rate in event-positive mice (**h**) was significantly increased after ISO (*white bars* CTL, *grey bars* TG; *p < 0.05 vs. CTL, ^#^ *p* < 0.05 vs. basal)

To test whether the increase in tCaR in single TG VCMs provokes events in a multi-cellular setting we recorded ECGs from TG vs. CTL mice under basal conditions and during acute isoproterenol challenge (ISO, i.p. injection). TG mice (age 19–21 weeks) showed on average more ventricular extrasystoles (VES) after ISO (Fig. 7c–e). Since TG mice develop atrial fibrillation, present in 86 % of all measured TG animals, we also investigated young mice (age 5–7 weeks) before the onset of AF to exclude AF itself as a contributor to the increase in VES. While the number of VES-positive mice was not different between groups the rate of VES/positive mice was 3.6 fold increased in TG mice compared to CTLs after ISO (Fig. 7f–h). Thus, the observations on the single cellular level are paralleled by an increased propensity to VES in TG mice.

### CREM-IbΔC-X is induced by chronic isoproterenol treatment

**Fig. 8:**
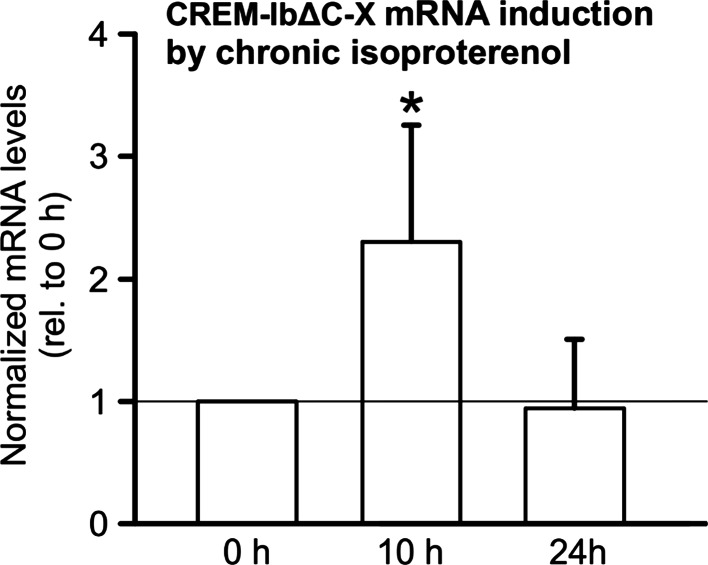
Relative mRNA expression levels of CREM-IbΔC-X on ventricular homogenates from CTL mice after isoproterenol treatment via osmotic minipumps. CREM-IbΔC-X was induced after 10 h isoproterenol treatment in CTL mice (CREM-IbΔC-X mRNA expression level normalized to *Hprt*, relative to 0 h, *n* = 11–12, *p < 0.05 vs. 0 h)

An analysis of the CREM gene revealed that similar or identical proteins could be translated by smICER, ICER and CREM-IbΔC-X transcripts (Suppl. Fig. 4). Since ICER and smICER can be induced by β-adrenergic stimulation, we examined whether this applies likewise for CREM-IbΔC-X. Quantification of CREM-IbΔC-X mRNA in ventricular homogenates from CTL mice treated with isoproterenol revealed a more than twofold induction of CREM-IbΔC-X mRNA after 10 h of treatment (Fig. 8).

## Discussion

Our results show that heart-directed expression of CREM-IbΔC-X, a CREM repressor isoform isolated from failing human hearts [25], evokes arrhythmogenic alterations in non-hypertrophied ventricular mouse cardiomyocytes including an increased incidence of transient-like spontaneous Ca^2+^ releases and early afterdepolarizations associated with an increased occurrence of ventricular extrasystoles.

The CREM repressor isoforms smICER, ICER and—as shown here—CREM-IbΔC-X are inducible by β-adrenergic stimulation. Though CREM-IbΔC-X, smICER and ICER [36] are only transiently induced by isoproterenol treatment using osmotic minipumps in mice, there is—at least in case of ICER—clear evidence that CREM repressors are sustainably induced in human heart failure and in mouse failing hearts induced by chronic pressure overload [10]. Since identical proteins (HIbI, HIbII) are translated from smICER and CREM-IbΔC-X mRNAs [25, 36] the cardiac overexpression of CREM-IbΔC-X should reflect to some extent consequences of the induction of short CREM repressor isoforms in the ventricle, and it may be speculated that the induction of CREM repressors in response to chronically elevated catecholamines contributes to the arrhythmogenic remodeling during the development of HF.

### CREM-IbΔC-X overexpression and spontaneous Ca^2+^ releases

The analysis of spontaneous Ca^2+^ releases in the current study showed an increased recruitment of VCMs with supra-threshold tCaR in TG, reflected by an increased number of event-positive cells, while the tCaR-frequency in event-positive cells was not different between groups. Determinants affecting the frequency of spontaneous Ca^2+^ releases are RyR2 phosphorylation and SR Ca^2+^ load. In TG atria hyperphosphorylation of RyR2 at S2814, leading to an enhanced SR Ca^2+^ leak and reduced SR Ca^2+^ load, has been linked to the development of AF in this model [7, 18]. In contrast, VCM’s SR Ca^2+^ load and ventricular RyR2 phosphorylation seemed to be unaltered between groups, which is in accordance with our observation of an unaltered event rate in event-positive VCMs.

The analysis of intracellular Ca^2+^ cycling revealed an accelerated Ca^2+^ transient decay in TG VCMs. We previously demonstrated that SERCA2a protein levels are increased in TG hearts [27] and confirmed this in the present study while the *Atp2a2*/SERCA2a mRNA level was unaltered between groups. The determination of the transport rates attributed to SERCA2a, NCX and PMCA identified not only an increased SERCA2a-mediated but also NCX-mediated Ca^2+^ transport rate. This went along with an increase in *I*_NCX_ and NCX1 protein but not mRNA levels in TG VCMs and hearts. The cardiac NCX generates an inward current by extruding 1 Ca^2+^ in exchange for 3 Na^+^ ions in its forward mode [5]. Thus, spontaneous Ca^2+^ waves will result in a membrane depolarization during Ca^2+^ extrusion by the NCX. An enhanced NCX transport capacity due to increased NCX1 protein levels actuates the extrusion of the same amount of Ca^2+^ in a shorter time which will lead to a more pronounced depolarization sufficiently large to trigger an AP. This “spontaneous” AP in turn will trigger a synchronized Ca^2+^ induced Ca^2+^ release visible as a Ca^2+^ transient. As a clear indication of this mechanism almost every detectable tCaR in our experiments was preceded by a Ca^2+^ wave, and the ratio tCaR/all spontaneous Ca^2+^ releases was increased in TG VCMs. This view is supported by two studies demonstrating that overexpression of NCX1 in a non-failing mouse model leads to an increased translation of sCaRs into DADs and spontaneous APs [32] and that heterozygous NCX1-knockout suppresses DADs and EADs [6].

In several models of hypertrophy and HF NCX expression or function is increased in face of reduced SERCA2a [37] and also in human failing hearts Ca^2+^ reuptake is usually impaired and SERCA2a protein levels mostly decreased [39]. However, in a mouse model with aortic banding NCX1 and SERCA2a were at first both upregulated during compensated hypertrophy before SERCA2a declined over time [14]. An increase in SERCA2a protein and function as observed in TG VCMs might have an antiarrhythmic effect. Increasing SERCA2a function by phospholamban-inactivation has been shown to break arrhythmogenic Ca^2+^ waves [4]. SERCA2a gene transfer in a HF model led to the reduction of Ca^2+^ alternans, reduced RyR2 phosphorylation and the reduced inducibility of ventricular arrhythmias [8]. Thus, it can be assumed that without the increase in SERCA2a the TG ventricular phenotype would be much more pronounced also with regard to the unaltered RyR2 phosphorylation.

### CREM-IbΔC-X overexpression and AP prolongation

In rodents L-type Ca^2+^ currents are relative small and the AP plateau phase can almost entirely be due to *I*_NCX_ [5] which was also demonstrated in a mouse model with cardiac-specific NCX1 overexpression [32]. Consequently, we found the ventricular AP prolonged in TG vs. CTLs along the increase in *I*_NCX_ and NCX1 protein levels while *I*_Ca,L_ was unaltered between groups. Regularly AP prolongation in models of HF goes along with the reduction of potassium currents—above all the transient outward current *I*_to_ [30, 39]. The *I*_to_ underlying channel subunits in rodents are the pore forming subunits Kv4.3, Kv4.2 and the accessory subunit KChIP2 [12]. In addition to the increase in *I*_NCX_, *I*_K,total_ peak amplitude was decreased in TG VCMs besides reduced Kv4.2 mRNA and KChIP2 protein levels whereas Kv4.2 protein was decreased at least in tendency in the observed samples. The decrease in *I*_to_ will additionally contribute to the observed AP prolongation in TG VCMs. However, since the APD_50_ at the same time was only increased by tendency in TG we speculate that this contribution is weaker compared to the increase in *I*_NCX_. In this context the increase of *Kcnb1*/Kv2.1 mRNA might point to a compensatory upregulation of *I*_kslow2_ [42] in TG VCMs to balance depolarizing and repolarizing currents [41] limiting AP prolongation in the late repolarization phase [40].

AP prolongation facilitates the occurrence of early afterdepolarizations (EADs) during AP repolarization and is a characteristic alteration of cardiomyocytes in animal and human failing hearts [39]. Indeed, we observed an increased propensity to EADs in TG VCMs as another arrhythmogenic consequence of the increase in *I*_NCX_ and the reduction of *I*_to_ in this model.

### Transcriptional regulation mediated by CREM repressor isoforms

The upregulation of NCX1 protein levels along with unaltered *Slc8a1* mRNA levels in TG cannot be explained by a direct transcriptional repression via CREM-IbΔC-X. Reportedly, NCX is upregulated by β_1_-AR stimulation via a CaMKII/AP-1 signaling pathway independent of CREB activation [21] despite the presence of CREs in the *Ncx1* promoter [22]. The β_1_-AR density has been found increased in TG ventricular homogenates [27] while in hearts of CREM KO mice the β_1_-AR density is decreased [26]. This underscores an important role of CREM repressors in β-adrenoceptor regulation. Consequently, the increased β_1_-AR density reported in TG ventricles may contribute to the NCX upregulation in TG VCMs.

The overexpression of CREM-IbΔC-X results in a reduction of *Kcnd2*/Kv4.2 mRNA along a tendency to reduced Kv4.2 protein levels in VCMs compared to controls. In our previous study we showed that cardiomyocyte-specific inactivation of the cAMP-dependent transcriptional activator CREB leads to AP prolongation due to an *I*_to_ reduction likewise along a decrease of *Kcnd2* mRNA and Kv4.2 protein in VCMs [34]. Our own results are supported by a recent study that postulates ICER as a repressor of *Kcnd2*/KV4.2/*I*_to_ by repressing miR-1 [28]. Hence, data from three independent mouse models strongly suggest that inactivation or repression of cAMP-dependent transcription leads to *I*_to_ remodeling.

### In vivo consequences of the observed arrhythmogenic alterations mediated by CREM-IbΔC-X

In TG mice with AF (19–21 weeks) and before the onset of AF (5–7 weeks) we observed a noticeable increase in the occurrence of VES especially after acute challenging with isoproterenol. The CREM mediated alterations seemed to increase above all the number of VCMs with tCaRs and EADs. This could be relevant when focusing on VESs. There has to be a critical number of cardiomyocytes with synchronized spontaneous events to generate a sufficient current source for the initiation of an ectopic trigger which may result in the initiation of cardiac arrhythmia [33]. β-adrenergic stimulation, on the other hand, has been shown to lead to spatio-temporal synchronization of spontaneous Ca^2+^ releases [29]. Consequently, the critical number of cardiomyocytes with synchronized spontaneous events required to generate a sufficient current source for the initiation of an ectopic trigger should be increased in TG mice during β-adrenergic stimulation. This may affect both the VES-frequency in susceptible mice as well as the general occurrence of VESs as was observed in the older TG mice, where the increased ratio VES/mouse vs. CTL during acute isoproterenol challenge (Fig. 7c) resulted from an increase in the number of VES-positive mice (Fig. 7d) and a strong tendency to an increased VES rate in VES-positive mice (Fig. 7e). Though VESs are common findings in ECGs it has recently been assessed that the frequent occurrence of VESs is associated with a substantial increase in the relative risk for sudden cardiac death in the general population in human [3].

## Conclusions

In summary, transgenic expression of CREM-IbΔC-X in cardiac tissue led to arrhythmogenic alterations in ventricular cardiomyocytes which could largely be attributed to an increase in *I*_NCX_. The arrhythmogenic alterations on the single cellular level were associated with an increased propensity to VESs in TG mice underlining the in vivo relevance of our findings. Since CREM-IbΔC-X is inducible by β-adrenergic stimulation and may be considered representative for other CREM repressor isoforms our results point to a role of cAMP-inducible inhibition of CRE-dependent transcription in the formation of an arrhythmogenic substrate during the development of chronic heart disease.

## Electronic supplementary material

Supplementary material 1 (DOCX 1100 kb)
