# Supplementary material for: Cardiac expression of the CREM repressor isoform CREM-IbΔC-X in mice leads to arrhythmogenic alterations in ventricular cardiomyocytes
Source: Basic Res Cardiol. 2016 Jan 27;111:15. doi: 10.1007/s00395-016-0532-y (PMC4729809; doi:10.1007/s00395-016-0532-y)

Cardiac expression of the CREM repressor isoform CREM-IbΔC-X in mice leads to arrhythmogenic alterations in ventricular cardiomyocytes

**Supplemental material**

**Supplemental methods:**

**Isolation of ventricular cardiomyocytes:**

Ventricular cardiomyocytes from adult mice were isolated as reported before [9, 11]. Mice were sacrificed by CO_2_ asphyxia. Hearts were explanted and immediately attached to a modified Langendorff apparatus for retrograde perfusion with respective buffers at 37°C and 2.5 ml/min. After perfusing the heart with perfusion buffer for 5 minutes (perfusion buffer: 113 mM NaCl, 4.7 mM KCl, 0.6 mM KH_2_PO_4_, 0.6 mM Na_2_HPO_4_·2H_2_O, 1.2 mM MgSO_4_·7H_2_O, 12 mM NaHCO_3_, 10 mM KHCO_3_, 30 mM taurine, 10 mM HEPES, 10 mM butanedione monoxime, 2 g/L glucose, pH = 7.45) collagenase (Worthington, type 2, 200 U/ml), protease (Sigma–Aldrich, type XIV, 0.33 U/ml) and 12.6 µM CaCl_2_ were added to digest the tissue for 6 minutes. Thereafter, cardiomyocytes were released by dispersing the digested ventricular tissue in 2.5 ml digestion buffer. To inactivate enzymes perfusion buffer supplemented with newborn calf serum (NCS) was added to the suspension (final concentration 5% NCS). After 10 minutes the supernatant was discarded, cells were resuspended in 10 ml perfusion buffer supplied with 5% NCS, filtered through a nylon gaze and gently centrifuged (42 g, 1 min). The resulting cell pellet was resuspended in 10 ml perfusion buffer and the external Ca^2+^ concentration was elevated stepwise to a final concentration of 1 mM (steps: 50 µM, 100 µM, 200 µM, 500 µM, 1 mM). After each of these five steps cells were allowed to adapt to Ca^2+^ elevation for at least 5 minutes.

**Patch Clamp recordings:**

APs and membrane currents were recorded in whole cell configuration using the perforated patch technique with amphotericin B [8]. Calcium-tolerant, rod-shaped and clearly striated ventricular cardiomyocytes were selected randomly for electrophysiological studies at room temperature (22–24°C). Patch pipettes were pulled (P97; Sutter, Novato, CA, USA) from borosilicate glass capillaries (Science Products, Hofheim, Germany) with a 1.5-mm diameter. Pipette resistances were 3–5 MΩ when filled with the pipette solutions. Recordings were performed using an EPC 800 Patch Clamp Amplifier (HEKA Elektronik, Lambrecht, Germany) or a SEC-05X discontinuous single-electrode voltage-clamp amplifier (NPI, Tamm, Germany) and PatchMaster Software (HEKA Elektronik, Lambrecht, Germany). Cell capacitance was determined by ramp pulses before capacitance compensation. Cell capacitance and series resistance were carefully compensated before recording start (EPC800). Recordings with series resistance >15 MΩ were discarded.

**APs** were evoked by injecting suprathreshold current pulses in current-clamp mode at a 2 Hz stimulation frequency. **Potassium currents** (I_K,total_) were evoked by a single step protocol from −40 to +60 mV for 25 s from a holding potential (HP) of −80 mV to achieve complete inactivation of transient I_K,total_ components according to Liu et al. [3]. Na^+^ currents were suppressed by applying a brief prepulse to −40 mV (10 ms) before the test pulse. In both experiments, the following solutions were used (in mM): pipette solution: 5 NaCl, 90 KCl, 35 KOH, 2.5 MgATP, 1 EGTA, and 5 HEPES pH 7.4; and extracellular solution 135 NaCl, 4 KCl, 1 MgCl_2_, 1.8 CaCl_2_, 0.33 NaH_2_PO_4_, 10 HEPES, and 10 glucose pH 7.4. L-type Ca^2+^ currents were blocked by nifedipine (2 μM) when potassium currents were recorded. **L-type Ca^2+^ currents (I_Ca,L_)** were evoked by a step protocol from −40 to +65 mV (Δ5 mV, 400-ms duration) following a 200 ms prepulse to −40 mV to inactivate T-type Ca^2+^ and Na^+^ currents (1 Hz stimulation frequency). Here the pipette solution was composed of the following (in mM): 120 CsCl, 4 MgCl_2_, 4 Na_2_ATP, 10 EGTA, and 10 HEPES pH 7.2. The extracellular solution contained the following (in mM): 135 NaCl, 3 CsCl, 1 KCl, 1.8 CaCl_2_, 1 MgCl_2_, 10 HEPES, and 10 glucose pH 7.4. The **NCX current** **(I_NCX_)** was recorded as described [7] using AP pipette and extracellular solution. Each cardiomyocyte was paced after establishing the whole cell configuration for 10-20 s at 1 Hz to ensure steady-state conditions. After a 10 s stimulation pause I_NCX_ was elicited by caffeine application (10 mM) directly to the cell by use of a fast application system (Warner Instruments, Hamden, CT, USA) while clamping the cardiomyocyte to a constant holding potential of -40 mV. Contamination of I_NCX_ with Ca^2+^-activated I_Cl_ was avoided by DIDS (0.1 mM). Peak amplitudes of membrane currents were normalized to cell capacitance and then plotted against test potentials to obtain respective current-voltage (I-V) relationships.

**Calcium imaging:**

Isolated ventricular cardiomyocytes were loaded with the Ca^2+^ indicators Fluo-4/AM (2-4µM) or Indo-1/AM (9 µM) (Molecular Probes®, Thermo Fisher Scientific, Waltham, MA, USA) for 10 min at room temperature. After incubation cells were placed in a pacing chamber on the stage of an inverted microscope (Diaphot 200 or Eclipse Ti-S, Nikon, Tokyo, Japan). After 5 min resting in Tyrode’s solution (140 mM NaCl, 5.8 mM KCl, 0.5 mM KH_2_PO_4_, 0.4 mM Na_2_HPO_4_ x2H_2_O, 0.9 mM MgSO_4_ x7H_2_O, 10 mM HEPES, 10 mM glucose, 2 mM CaCl_2_, pH 7.3 adjusted with NaOH) and an additional phase of 5 min perfusion with Tyrode’s solution, cardiomyocytes were field-stimulated for another 10 min by an electrical stimulation system (Myopacer, Ionoptix, Milton, MA, USA or CS Type 223, Hugo Sachs Elektronik-Harvard Apparatus GmbH, March-Hugstetten, Germany) before recording start. Fluorescence was measured with excitation wavelength at 488 nm (Fluo-4) or 340 nm (Indo-1) and the emitted fluorescence (Fluo-4: 522 nm, Indo-1: 405/495) was detected by photomultipliers (RatioMaster™, PTI, Birmingham, USA or Myocyte Calcium and Contractility System, Ionoptix, Milton, MA, USA). Background acquisition was performed with a cell free area of the suspension in the pacing chamber and was automatically subtracted from following measurements by FeliX computer software (Photon Technology International, North Edison, NJ, USA) or Ionwizard software (Ionoptix, Milton, MA, USA). **Ca^2+^ transients** were recorded at 0.5 Hz stimulation with Indo-1/AM. Transients from at least 10 cardiomyocytes were recorded under basal conditions. **Fractional Ca^2+^ reIease and SR Ca^2+^ load** were determined using Indo-1/AM by rapid caffeine application (10 mM) after a 10 s pacing pause following a 1 Hz pre-stimulation phase. SR Ca^2+^ load was estimated as the amplitude of the caffeine induced transient and the fractional release was calculated from the amplitude of electrically stimulated transients during the prestimulation phase in relation to caffeine induced transients. **Ca^2+^ transport by Na^+^/Ca^2+^ exchanger (NCX), sarcoplasmic reticulum Ca^2+^-ATPase (SERCA2a) and plasmalemmal Ca^2+^-ATPase (PMCA)** was estimated using Fluo-4/AM from the rate constants (r) of single exponential curves fitted to the decay of electrically and caffeine-evoked Ca^2+^ transients as described [1, 5]. We chose Fluo-4/AM as Ca^2+^ sensitive dye in this experimental series to achieve a better signal-to-noise ratio for a more precise assessment of decay kinetics by single exponential curve fitting at which a ratiometric measurement (Indo-1) is not necessarily required. Single ventricular cardiomyocytes were randomly chosen and subjected to one of the following two protocols: (1) Perfusion with normal Tyrode’s solution and field-stimulation (1Hz/ 20 s) was followed by a pacing-pause (10 s) and rapid application of caffeine (10 mM, dissolved in Tyrode’s solution). (2) Perfusion with normal Tyrode’s solution and field-stimulation (1Hz/ 20 s) was followed by a pacing-pause (60 s) in which the chamber was perfused with 0-Na^+^-0-Ca^2+^-Tyrode’s solution (140 mM LiCl, 4 mM KCl, 1 mM MgCl2, 5mM HEPES, 10mM Glucose, 10mM EGTA; pH 7.4 adjusted with LiOH) and then the cells were exposed to caffeine (10 mM, dissolved in 0-Na^+^-0-Ca^2+^-Tyrode’s solution). At the end of both protocols cells were perfused with normal Tyrode’s solution and post-treated (30 s/ 1 Hz) in order to rate their state of health. Curve fitting was performed with OriginPro (OriginLab, Northhampton, MA, USA) and the mean of six technical replicates per protocol and preparation was used in the statistical analysis. The decay rate of an electrically stimulated Ca^2+^ transient reflects the rate of combined transport by SERCA2a, NCX and PMCA (r1=r_SERCA_ +r_NCX_ +r_PMCA_). Caffeine induced transients reflect the combined transport by NCX and PMCA (r2= r_NCX_ +r_PMCA_) whereas PMCA alone is responsible for the caffeine induced transient decay during perfusion with 0-Na^+^-0-Ca^2+^-Tyrode’s solution (r3=r_PMCA_). The individual transport rates have therefore been estimated by subtraction: r_SERCA_=r1-r2, r_NCX_=r2-r3 and r_PMCA_=r3.

**Morphometric VCM analysis:**

Ventricular cardiomyocytes freshly isolated for single cell studies were transferred into a custom made cell chamber mounted on the motorized stage of an inverted microscope (Eclipse Ti-E, Nikon, Tokyo, Japan). After VCMs have sedimented by gravity a wide field-of-view image was generated by stitching 5 x 5 adjacent frames taken automatically (10x magnification) and consecutively using NIS Elements AR software (Nikon, Tokyo, Japan). In this image at least 50 neighboring, rod-shaped and clearly striated VCMs were selected for morphometric analysis. The longitudinal cell axis was taken as VCM length. VCM surface area was measured by tracing cell edges with a polygon function (NIS Elements BR software, Nikon, Tokyo, Japan). Cell width was estimated by dividing the individual cell surface area by the respective cell length (Excel 2010, Microsoft, Washington, USA).

**Quantitative real-time RT PCR:**

Quantitative real-time RT-PCR was performed on ventricular homogenates and ventricular cardiomyocytes. Relative expression ratios of mRNAs were derived after the ΔΔC_T_ method using REST software [4, 6, 12] as described previously [9, 10]. The following primer pairs have been used for quantitative real-time RT-PCR:

| **Gene** | **Primer Pairs** |
| --- | --- |
| *Kcnip2* (KChIP2) | F-5’-gacatgatgggcaagtacacc-3’  R-5’-acgccgtccttgtttctgt-3’ |
| *Kcna4* (Kv1.4) | F-5’-gaagaaggggtcaaggaatc-3’  R-5’-tggcaggtggagagaacaat-3’ |
| *Kcna5* (Kv1.5) | F-5’-catcaaggaagaggagaagc-3’  R-5’-gaaaggacaccagagccact-3’ |
| *Kcnb1* (Kv2.1) | F-5’-ccaccagattctcccacagt-3’  R-5’-gctctccacgaagaaaccag-3’ |
| *Kcnd2* (Kv4.2) | F-5’-ctgctcacggagacacaaaa-3’  R-5’-cggctgttggatagtggagt-3’ |
| *Kcnd3* (Kv4.3) | F-5’-tgtacgaacctccaccatca-3’  R-5’-agtggctggacagagaagga-3’ |
| *Atp2a2* (SERCA2a) | F-5’-ctgtggagacccttggttgt-3’  R-5’-cagagcacagatggtggcta-3’ |
| *Hprt* (hypoxanthine-guanine phosphoribosyltransferase) | F-5’-atgagcgcaagttgaatctg -3’  R-5’-ggacgcagcaactgacatt -3’ |

**Immunoblotting:**

Ventricular homogenates were prepared and quantitative immunoblotting performed as previously described ^9^. The following antibodies have been used: Kv4.2 (APC-023, 1:200, Alomone, Jerusalem, Israel), KChIP2 (sc-256851, 1:200, Santa Cruz Biotechnology, Dallas, TX, USA), CSQ (PA1-913, 1:2500, Thermo Fisher Scientific, Waltham, USA), GAPDH (ab9485, 1:10000, Abcam, Cambridge, UK), NCX1 (R3F1, 1:1000, Swant, Marly, Switzerland), RyR (mouse monoclonal antibody: 1E9 [13], 1:1000), RyR Ser2814 (A010-31AP, 1:2000, Badrilla, Leeds, UK), RyR Ser2808 (A010-30AP, 1:1000, Badrilla, Leeds, UK), SERCA2a (mouse monoclonal antibody: 2A7A1 [2], 1:1000).

**Electrocardiographic measurements**

ECG recordings were performed on old (19-21 weeks of age) and young mice (5-7 weeks of age). After inhalation anesthesia with an isoflurane-nitrous oxide mixture mice were positioned supine on a 37°C heating plate and 5 subcutaneous limb electrodes were placed after loss of toe pinch reflex. For electrocardiogram recordings electrodes were connected over an external biological amplifier (Dual Bio Amp, ADInstruments, Dunedin, New Zealand) to a data acquisition unit (PowerLab 2/20, ADInstruments, Dunedin, New Zealand), which recorded lead I and II and calculated lead III, aVR, aVL und aVF. The protocol included a 10 min recording-phase under basal condition followed by an additional 10 min (old mice)/ 20 min (young mice) recording-phase after intraperitoneal injection of isoproterenol (2 mg/kg bodyweight). QRS complex duration was determined in lead I using LabChart 7 Pro software (ADInstruments, Dunedin, New Zealand). Thereby a QRS complex was defined as a ventricular extrasystole (VES) on condition that 1) a previous p-wave was absent and that 2) the QRS complex duration was longer than the mean plus twofold standard deviation of three previous regular QRS complexes.

References

1. Choi HS, Eisner DA (1999) The role of sarcolemmal Ca 2+ -ATPase in the regulation of resting calcium concentration in rat ventricular myocytes. J Physiol 515: 109–118. doi: 10.1111/j.1469-7793.1999.109ad.x

2. Jones LR, Zhang L, Sanborn K et al. (1995) Purification, Primary Structure, and Immunological Characterization of the 26-kDa Calsequestrin Binding Protein (Junctin) from Cardiac Junctional Sarcoplasmic Reticulum. J Biol Chem 270: 30787–30796. doi: 10.1074/jbc.270.51.30787

3. Liu J, Kim K, London B et al. (2011) Dissection of the voltage-activated potassium outward currents in adult mouse ventricular myocytes: I(to,f), I(to,s), I(K,slow1), I(K,slow2), and I(ss). Basic Res Cardiol 106: 189–204. doi: 10.1007/s00395-010-0134-z

4. Livak KJ, Schmittgen TD (2001) Analysis of relative gene expression data using real-time quantitative PCR and the 2(-Delta Delta C(T)) Method. Methods 25: 402–408. doi: 10.1006/meth.2001.1262

5. Maczewski M, Mackiewicz U (2008) Effect of metoprolol and ivabradine on left ventricular remodelling and Ca2+ handling in the post-infarction rat heart. Cardiovasc Res 79: 42–51. doi: 10.1093/cvr/cvn057

6. Pfaffl MW (2002) Relative expression software tool (REST©) for group-wise comparison and statistical analysis of relative expression results in real-time PCR. Nucleic Acids Res 30: 36e. doi: 10.1093/nar/30.9.e36

7. Pott C, Muszynski A, Ruhe M et al. (2012) Proarrhythmia in a non-failing murine model of cardiac-specific Na+/Ca 2+ exchanger overexpression: whole heart and cellular mechanisms. Basic Res Cardiol 107: 247. doi: 10.1007/s00395-012-0247-7

8. Rae J, Cooper K, Gates P et al. (1991) Low access resistance perforated patch recordings using amphotericin B. J Neurosci Methods 37: 15–26. doi: 10.1016/0165-0270(91)90017-T

9. Schulte JS, Seidl MD, Nunes F et al. (2012) CREB critically regulates action potential shape and duration in the adult mouse ventricle. Am J Physiol Heart Circ Physiol 302: H1998-2007. doi: 10.1152/ajpheart.00057.2011

10. Seidl MD, Nunes F, Fels B et al. (2014) A novel intronic promoter of the Crem gene induces small ICER (smICER) isoforms. FASEB J 28: 143–152. doi: 10.1096/fj.13-231977

11. Tekook MA, Fabritz L, Kirchhof P et al. (2012) Gene construction, expression and functional testing of an inotropic peptide from the venom of the black scorpion Hottentotta judaicus. Toxicon 60: 1415–1427. doi: 10.1016/j.toxicon.2012.10.008

12. Vandesompele J, Preter K de, Pattyn F et al. (2002) Accurate normalization of real-time quantitative RT-PCR data by geometric averaging of multiple internal control genes. Genome Biol 3: RESEARCH0034.1. doi: 10.1186/gb-2002-3-7-research0034

13. Zhang L, Kelley J, Schmeisser G et al. (1997) Complex Formation between Junctin, Triadin, Calsequestrin, and the Ryanodine Receptor. Proteins of the cardiac junctional sarcoplasmic reticulum membrane. J Biol Chem 272: 23389–23397. doi: 10.1074/jbc.272.37.23389

**Supplemental figure legends:**

**Supplemental Fig. 1:**

Time distribution of tCaRs recorded in the pacing pause grouped into 3 phases 0-30, 30-60 and 60-90 s. Note that tCaRs tend to occur earlier following an increased pacing rate (2Hz) and in general earlier in TG VCMs (arrows).

**Supplemental Fig. 2:**

(A) Representative immunoblots of SERCA2a and CSQ. SERCA2a protein levels were increased (B) in CREM TG vs. CTL ventricular homogenates whereas *Atp2a2*/SERCA2a mRNA levels (C) were unaltered between groups (CREM TG vs. CTL, SERCA2a protein level normalized to CSQ: 1.45±0.16* vs. 0.99±0.10, *p<0.05 vs. CTL; relative *Atp2a2* mRNA expression level were normalized to *Hprt*, mean[std. error]: 1.08[0.790 - 1.443], n=12)

**Supplemental Fig. 3:**

VCM size and capacity. (A) VCM length, (B) width and (C) area were not different between groups as determined by wide-field microscopy. Boxplots reflect the mean values of 14 animals per group which have been calculated from 50 VCMs/animal. Accordingly, VCM capacity values averaged from the different patch clamp measurements were unaltered between TG and CTL. Grey boxes: TG, white boxes: CTL.

**Supplemental Fig. 4:**

Scheme of the *Crem* gene structure, transcripts of *ICER*, *smICER* and *CREM-IbΔC-X* and possibly translated proteins. Different promoter utilization and alternative splicing leads to the transcription of different short CREM repressor isoforms, from which up to 4 different proteins may be translated. (P1-6: promoters, Boxes: exons, ATGs: possible translation start sites). For clarity transcripts and proteins containing the Ia-exon are omitted.


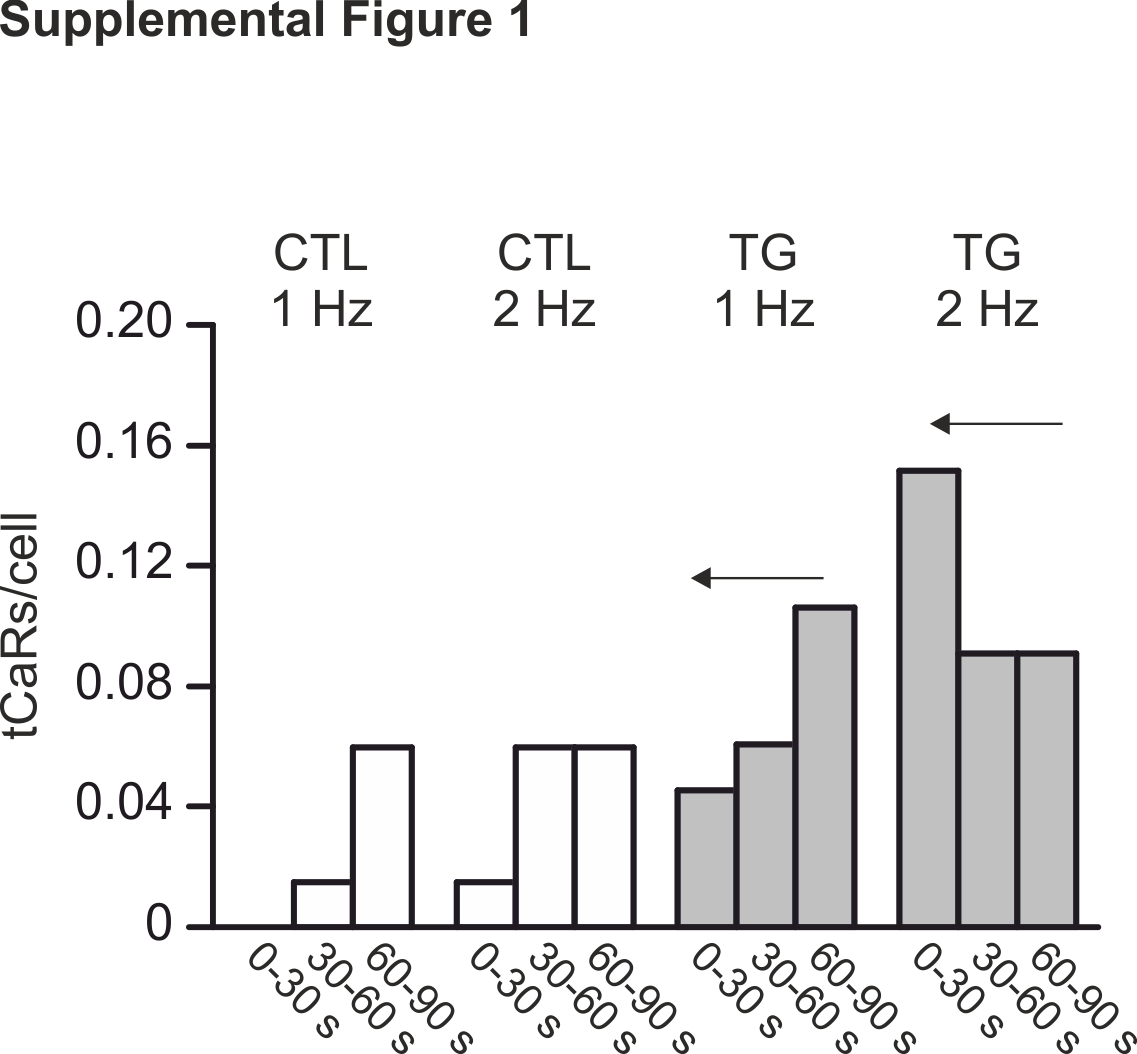


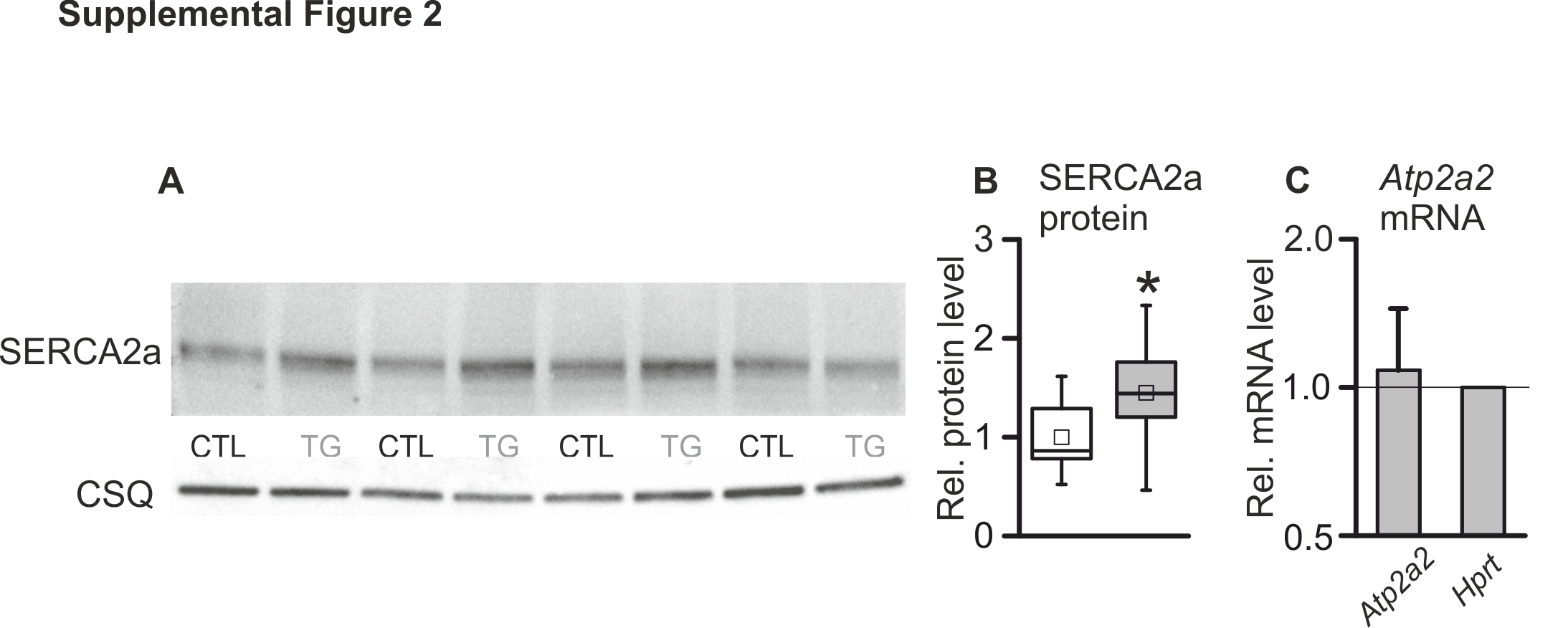


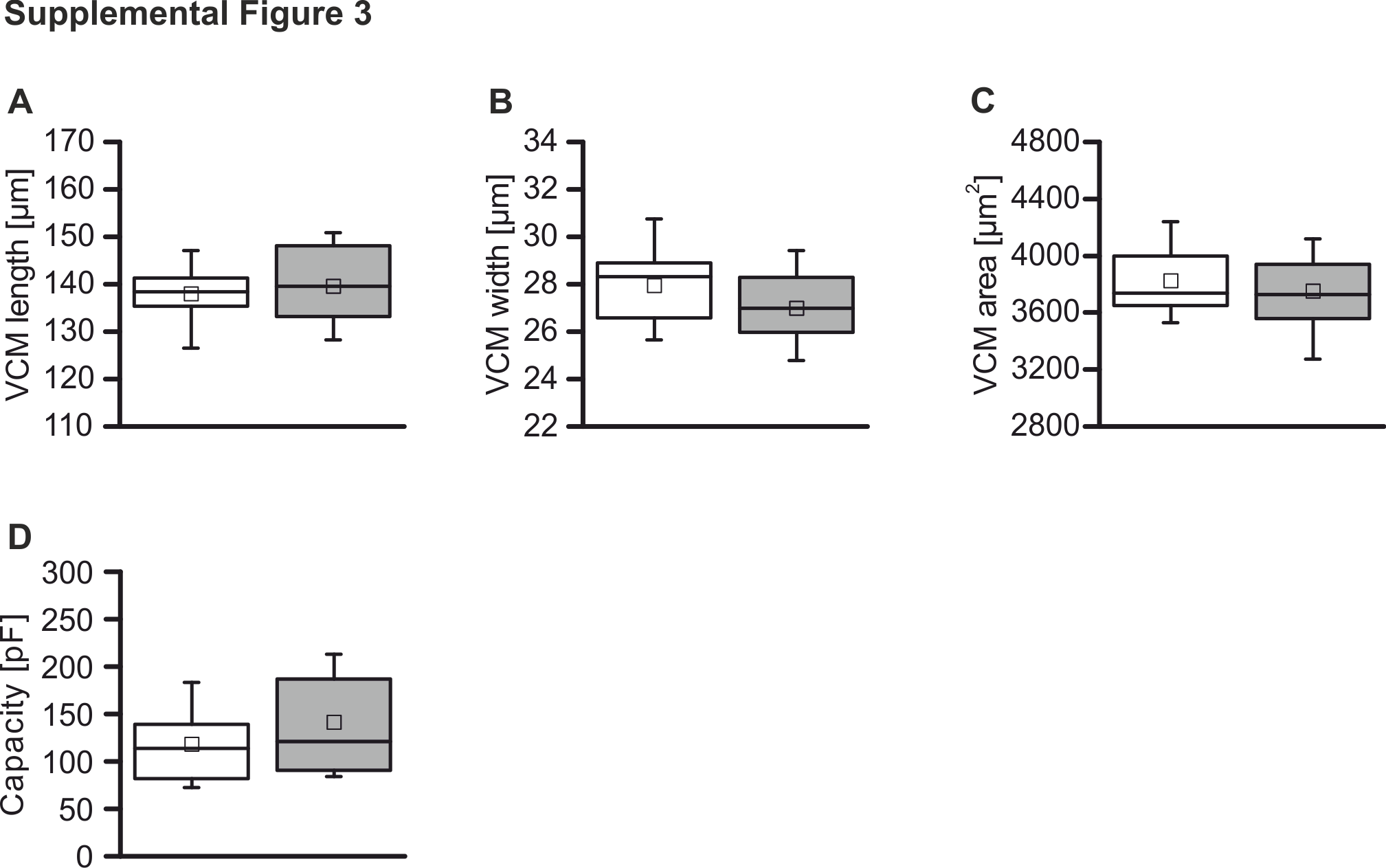


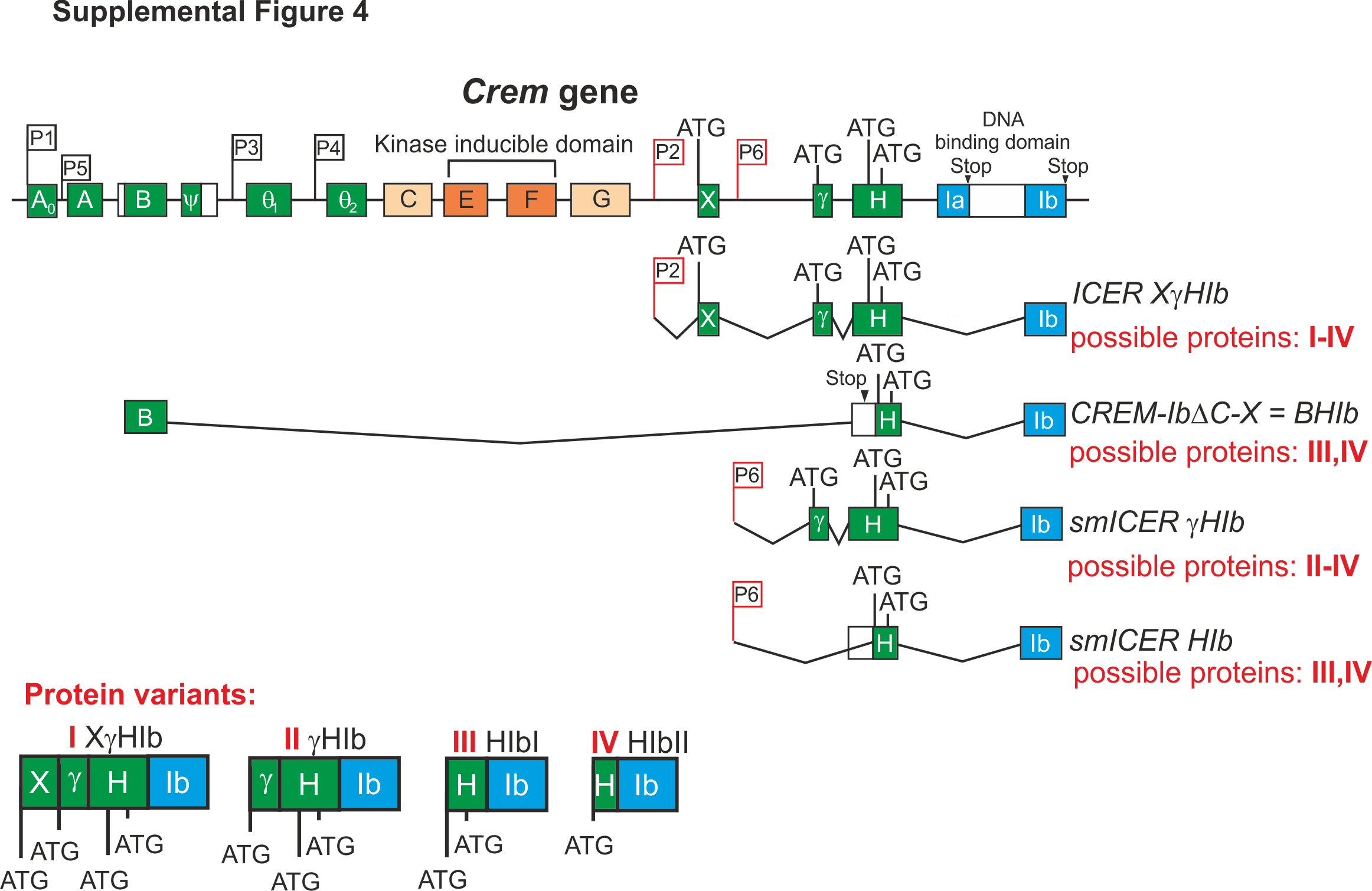

Supplement: Supplementary file 1 — Supplementary material 1 (DOCX 1100 kb) [file 395_2016_532_MOESM1_ESM.docx]
